# Data from a public participation GIS survey on the everyday active travel experiences of residents from five European cities

**DOI:** 10.1016/j.dib.2026.112981

**Published:** 2026-06-15

**Authors:** Rory Taylor, Silviya Korpilo, Elias Willberg, Kamyar Hasanzadeh, Robert Klein, Kerli Müürisepp, Anton Stahl Olafsson, Tuuli Toivonen

**Affiliations:** a Digital Geography Lab, Department of Geosciences and Geography, University of Helsinki, PL 64, Gustaf Hällströmin katu 2, 00014 Helsinki, Finland; b Finnish Environment Institute, Latokartanonkaari 11, 00790 Helsinki, Finland; c Helsinki Institute of Sustainability Science, University of Helsinki, Yliopistonkatu 3, 00014 Helsinki, Finland; d Helsinki Institute of Urban and Regional Studies, University of Helsinki, Yliopistonkatu 3, 00014 Helsinki, Finland; e Department of Geosciences and Natural Resource Management, University of Copenhagen, Rolighedsvej 23, 1958 Frederiksberg C, Copenhagen, Denmark

**Keywords:** Active mobility, Street greenery, Everyday travel environment, Public participation GIS

## Abstract

This article describes two coupled datasets collected via an online Public Participation GIS (PPGIS) survey into respondent’s everyday active travel patterns, their perceptions and experiences of their travel environments, and how these change across different seasons (specifically summer and winter). The survey was distributed through Maptionnaire across five European cities: Helsinki Metropolitan Area (1114 respondents), Greater Copenhagen (1225 respondents), Greater London (1188 respondents), Greater Munich (855 respondents) and Las Palmas de Gran Canaria (582 respondents) between November 2023 and February 2024. The first dataset comprises 4,964 anonymised survey responses in CSV format, organised by city, that detail travel patterns, multi-sensory perceptions, and socio-demographic information across summer and winter. The second dataset is spatial, provided in shapefile format, and includes 8,048 mapped points of pleasant and unpleasant travel places along everyday travel routes and 1,241 points of places along those routes where respondents desire additional street greenery across the five study cities. Mapped variables include sensory aspects like aesthetics and soundscape, along with specific street greenery elements such as street trees and plant beds. Together, these datasets enable inter-city and seasonal comparisons that may be of value to researchers and urban planners working to understand the relationships between urban street environments and active travel behaviour of urban residents in different European cities.

Specifications Table

**Table:** 

Subject	Geography – Urban and transportation planning
Specific subject area	Spatio-temporal patterns of active travel experiences, perceptions and greenspace exposure within and between European cities.
Type of data	(i) Dataset: CSV file (ii) Dataset: Shapefile point data, accessible at [1] S. Korpilo, K. Hasanzadeh, R. Klein, E. Willberg, R. Taylor, K. Müürisepp, A.S. Olafsson, T. Toivonen, (2025). GREENTRAVEL Public Participation GIS survey on active mobility in Europe [dataset]. Zenodo v2, 2025. https://doi.org/10.5281/zenodo.17866393
Data format	Raw
Parameters for data collection	Internet based PPGIS survey distributed through Maptionnaire software.
Description of data collection	Data collected through a panel company and Danish Registry Data (Copenhagen only) using a random sampling approach across five European cities from late November 2023 to mid-February 2024. Study cities (*survey language indicated)*: -Helsinki Metropolitan Area *(Finnish and English)* -Greater London (*English)* -Munich (*German and English)* -Las Palmas de Gran Canaria *(Spanish and English)* -Greater Copenhagen (*Danish and English).* * *Greater Copenhagen required two sampling stages to meet participation number targets. The second stage occurred in late May and June 2024.
Data source location	*Primary institution:* University of Helsinki, Helsinki, Finland *Secondary institution*: University of Copenhagen, Copenhagen, Denmark
Data accessibility	Non-spatial survey data: With this article. Spatial data of mapped pleasant and unpleasant points and street greenery preferences along active travel routes: Korpilo, S., Hasanzadeh, K., Klein, R., Willberg, E., Taylor, R., Müürisepp, K., Olafsson, A., & Toivonen, T. (2025). GREENTRAVEL Public Participation GIS survey on active mobility in Europe [Data set]. Zenodo. https://doi.org/10.5281/zenodo.17866393
Related research articles	[2] S. Korpilo, K. Hasanzadeh, R. Klein, E. Willberg, R. Taylor, K. Müürisepp, A.S. Olafsson, T. Toivonen, Green and blue spaces matter for active mobility: Results from mapping perceived environmental exposure in five European cities, *Urban Forestry and Urban Greening* 113 (2025). doi:https://doi.org/10.1016/j.ufug.2025.129088 [3] E. Willberg, S. Korpilo, R. Klein, R. Taylor, K. Müürisepp, T. Kemppainen, T. Toivonen, ‘Universal or local preferences? The perceived importance of the urban environment on active travel behaviour in Europe across populations’, submitted to Journal of Transport Geography (unpublished) [4] K. Hasanzadeh, S. Korpilo, R. Klein, E. Willberg, A. Saarinen, J. Torkko, T. Toivonen, ‘Predicting perceived pleasantness of everyday active mobility environments’, submitted to *Cities* (unpublished).

## 1 Value of the Data

• These coupled survey datasets provide new insights into urban street environment, street greenery and active travel associations among urban residents across five European cities (Helsinki Metropolitan Area, Greater Copenhagen, Greater London, Munich and Las Palmas de Gran Canaria).
• The datasets allow for inter- and intra-city comparison of active travel experiences, perceptions and perceived environmental exposure in urban street environments.
• The datasets allow for season-sensitive analysis of these associations across summer and winter.
• These datasets can be useful to researchers working to understand the relationships between urban street environments and active travel behaviour of urban residents of European cities, as well as beneficial to policy-makers and urban planning practitioners seeking to improve the sustainability, biodiversity and wellbeing benefits of city streets.
• Specifically, the datasets can be applied, for example, in targeting street interventions to reduce unpleasant travel experiences as part of sustainable mobility policies, or in prioritising locations for adding vegetation as part of urban greening efforts in the study cities.

## 2 Background

This article describes two coupled datasets collected through the online Public Participation GIS (PPGIS) survey *Greentravel: A survey on your everyday active travel environment’¸* developed and distributed as part of the ERC funded project *GREENTRAVEL – Greener Urban Travel Environments for Everyone: From Measured Wellbeing Impacts to Big Data Analytics* (101044906) at the University of Helsinki.

This data article supports three research articles [[2], [3], [4]] linked with the GREENTRAVEL project that use the PPGIS survey data. The article aims to describe and improve access to the multi-city PPGIS survey data underpinning this research and facilitate its further use in future research and urban planning practice, including applications at the local scale within each of its five European study cites.

## 3 Data Description

The GREENTRAVEL survey sought participants’ responses into their everyday active travel patterns, what they perceived as important, how they experienced their travel environment, and how these perceptions and experiences change in different seasons (specifically, summer and winter). **Everyday active travel** was defined for survey participants as *non-motorised forms of travel such as walking or cycling that you use on a regular daily basis to get to a destination (e.g. go to work, school, shop, but excluding trips for leisure).*

The first dataset, *GREENTRAVEL_Survey_EverydayTravel_Data,* provided in .csv format in the Appendix, is composed of 4964 rows of data and includes anonymised participant responses to six questions about their everyday active travel patterns and their experiences across summer and winter. Each question was asked first for summer, then repeated for winter if the respondent indicated to a prompt asking if their answer would change in winter. Questions related to travel patterns include travel mode, travel choice, travel route motivation, the sensory aspects of pleasant and unpleasant travel experiences as well as questions relating to travel duration. There were 4964 survey respondents in total: Helsinki Metropolitan Area (1114 respondents), Greater Copenhagen (1225 respondents), Greater London (1188 respondents), Greater Munich (855 respondents) and Las Palmas de Gran Canaria (582 respondents). In addition, socio-demographic background information sought during the survey included perceived busyness in daily life, gender, age, household size, number of children in household, education, work status, mother tongue and income. An overview of the respondent demographics of the GREENTRAVEL survey is provided in Table 1. Overall, the survey response group was generally representative of the general population across age and gender in each study city, though the oldest groups (65+) experienced some under-representation.

**Table 1:** Socio-demographic characteristics of survey respondents.

	Greater Copenhagen (%)	Helsinki Metropolitan Area (%)	Greater London (%)	Munich (%)	Las Palmas (%)
*Perceived busyness (1 = not at all busy, 10 = extremely busy)*	***N= 580***	***N= 586***	***N= 485***	***N = 471***	***N = 275***
*Low busyness (1 to 4)*	26.0	33.1	16.3	7.4	13.8
*Moderate busyness (5)*	5.7	4.3	5.4	5.7	4
*High busyness (6 to 10)*	68.3	62.5	78.3	86.8	82.2
*Gender*	***N = 1091***	***N = 1028***	***N = 1023***	***N = 787***	***N= 542***
*Female*	53.4	53.1	48.9	41.4	53.5
*Male*	45.4	45.0	50.4	57.9	45.9
*Other*	0.4	1.2	0.3	0.3	0.6
*Age*	***N= 1082***	***N = 1027***	***N = 1023***	***N= 782***	***N=539***
*Young adult (18 to 34)*	43.8	32.0	31.5	43.9	41.0
*Middle-aged (35 to 64)*	41.8	57.3	52.5	55.8	56.0
*Older adults/elderly (65+)*	14.3	10.7	16.1	0.4	3.0
*Household size*	***N=898***	***N=763***	***N= 789***	***N=619***	***N=469***
*1*	12.0	19.8	11.4	15.5	5,3
*2*	41	38.7	31.7	32.3	29.6
*3*	16.8	15.5	17.5	21.2	23.9
*4*	18.2	13.5	17.5	11.6	19.8
*5+*	12.0	12.6	21.9	19.4	21.3
*Number of children in household*	***N = 895***	***N= 855***	***N = 871***	***N = 649***	***N= 470***
*0*	65.5	64.3	66.8	59.0	54.3
*1*	17.8	18.6	17.0	21.9	26.4
*2*	13.9	13.2	12.4	15.3	15.7
*3+*	2.9	3.9	3.8	3.9	3.6
*Education*	***N= 1062***	***N= 1003* **	***N= 1000* **	***N= 747* **	***N=522* **
*No formal schooling completed*	8.4	9.5	1.20	3.1	2.1
*Upper secondary education*	18.7	31.6	23.3	21.0	23.2
*Trade/Technical/* *vocational training*	15.7	10.0	13.3	33.7	38.1
*Bachelor’s degree*	24.3	25.1	39.6	18.9	26.1
*Master’s degree*	30.0	22.5	20.0	20.6	7.9
*Doctoral degree*	2.8	1.30	2.6	2.7	2.7
Work status*	***N= 1026***	***N= 1108***	***N= 1087***	***N = 744***	***N = 582***
*In full time paid work*	52.9	45.9	45.5	64.1	50.3
*In part time work*	11.4	10.5	12.3	12.9	9.3
Unemployed**	3.8	23.0	21.2	8.6	25.6
*Retired*	12.3	11.0	12.4	2.6	4.1
*Student*	23.0	25.0	20.2	13.7	22.7
*In community or military service*	0.3	0.0	0.1	0.1	1.0
*Permanently sick/disabled*	1.4	1.5	1.2	1.3	1.5
*Stay at home, looking* *after children or* *other persons*	0.5	1.0	3.1	1.6	1.4
*Income distribution*	***N= 1064***	***N=1012***	***N=1002***	***N=769***	***N=534***
*1^st^ quintile*	(< 7 500kr) 22.1	(<1 000 €) 13.4	(<1 000 £) 13.9	(<1 000 €) 10.8	(<500 €) 15.0
*2^nd^ quintile*	(7 500 to 15 000kr) 19.5	(1 000 to 2000 €) 25.6	(1 000 to 2000 £) 28.6	(1 000 to 2000 €) 17.4	(500 to 1 500 €) 44.4
*3^rd^ quintile*	(15 001 to 22 500kr) 24.8	(2 001 to 3000 €) 30.7	(2 001 to 3000 £) 25.9	(2 001 to 3000 €) 31.2	(1 501 to 2 500 €) 28.5
*4^th^ quintile*	(22 501 to 30 000kr) 17.8	(3 001 to 4000 €) 17.5	(3 001 to 4000 £) 16.9	(3 001 to 4000 €) 22.6	(2 501 to 3 500 €) 9.0
*5^th^ quintile*	(>30 000kr) 15.9	(> 4 000 €) 12.8	(> 4 000 £) 14.8	(> 4 000 €) 17.9	(> 3 500 €) 3.2
Mother tongue (top three most common)***	***N = 1058***	***N = 954***	***N = 864***	***N = 737***	***N = 525***
*1*	(Danish) 87.3	(Finnish) 95.6	(English) 92.2	(German) 89.55	(Spanish) 95.6
*2*	(English) 4.5	(English) 2.7	(Spanish) 2.4	(English) 10.2	(Italian) 2.3
*3*	(German) 1.9	(Swedish) 2.5	(Polish) 1.8	(Turkish) 3.8	(English) 1.5

*Survey participants could identify multiple work status options.

** Many participants identified as both unemployed and students (HMA: 15.7%; Greater London: 16.0%; Munich: 5.2%; Las Palmas: 14.8%; Greater Copenhagen: 0.2%).

***Survey participant could identify multiple mother tongue options.

A codebook to define each variable and aid interpretation of the data by users accompanies the dataset and is provided in the Appendix.

The second dataset, *GREENTRAVEL Public Participation GIS survey on active mobility in Europe,* provides a total of 8048 individually mapped points of pleasant and unpleasant places along everyday active travel routes identified by survey respondents across the five study cities. It additionally provides 1,241 mapped points of places along those routes to add street greenery identified by respondents across these cities. Table 2 summarises the division of these responses by study city. Pleasant and unpleasant categories were predefined for respondents (described further below), though an option for ‘other’ was included. Street greenery elements available for respondents to select from included street trees, shrubs, vertical greenery, plant beds and parklets, lawns and meadows, seasonal flowers and other. The data is provided in shapefile format and is accessible through its Zenodo repository [1]. Refer to the PPGIS survey provided in the Appendix for the mapping prompts provided to respondents.

**Table 2:** Number of mapped points by study city in the GREENTRAVEL PPGIS survey.

Study city	Pleasant places	Unpleasant places	Places to add street greenery
Greater Copenhagen	1545	1090	411
Helsinki Metropolitan Area	1222	753	307
Greater London	1053	445	201
Munich	751	376	122
Las Palmas	552	261	200

The two datasets provided and described here are linked by a unique ID for each survey respondent (R_ID).

## 4 Experimental Design, Materials and Methods

An online PPGIS survey was distributed through Maptionnaire from November 2023 to February 2024 among adult respondents residing in five European cities: Helsinki Metropolitan Area, Greater London, Greater Copenhagen, Munich and Las Palmas de Gran Canaria. These cities were selected as study sites due to their differences in climatic conditions and diversity in population size, urban morphology and the popularity of active travel among their residents. The survey was distributed using a random sampling approach by the panel company Taloustutkimus Oy, with the exception of Greater Copenhagen, where respondents were sampled directly through Danish Registry Data as the researchers had the access to this data. A second round of sampling was required in Greater Copenhagen in late May and June 2024 due to insufficient responses in the first sampling round. During the sampling, the panel company and responsible researchers monitored the sample to ensure representativeness across population groups.

Survey structure and questions were standardised, and consistent across each study city. The mobility-related questions were informed by travel behaviour literature and typical travel surveys (e.g., [5]) while the questions on pleasant and unpleasant experiences were adapted from previous literature on personal exposure and multisensory perceptions of environmental quality [6,7], and [8]. The survey also included standardized pan-European demographic survey questions derived from European Social Survey [9] including: gender, age, mother tongue language, household structure, number of children, employment status, education and income. The survey was provided in both English and the local language of each city, and implemented in accordance with the principles of informed consent and in compliance with the *EU General Data Protection Regulations*. The translation of the survey from English into each local language was carried out by researchers who were native speakers of the target language, and fluent in English. Prior to translation, they were briefed on the intended meanings of the survey terminology to ensure consistency across languages.

The survey was organised into two complementary sections. Section one was directed towards understanding the everyday travel patterns of respondents in both summer and winter (Willberg et al., under review). To explore whether there were differences across seasons, respondents were firstly prompted to answer each survey question based on their experiences in summer, before identifying if their responses would be different in winter. If different, the question would be repeated for winter in a pop-up window. The questions, variables and response types for section one of the survey are provided in Table 3 below.

**Table 3:** Everyday active travel patterns and experiences - variable categories.

	Survey question	Variable	Response type
Travel mode	**Question 1 (Q1).** In the SUMMER, which travel modes do you use most in your everyday travel (e.g. to go to work, school, shop, but excluding trips for leisure)? Please rank up to three options according to time spent. * Does this change over WINTER?	• Walking • Cycling • Electric bike • Electric scooter • Car • Public Transport	*Multiple choice*
Travel choice	**Question 2 (Q2)**. Thinking about SUMMER, to what extent do you agree or disagree with the following statements about your everyday travel choices? * Does this change over WINTER?	• The environment along my route affects whether I travel or not • The environment along my route affects my transport mode choice (e.g. walk, cycle, car or public transport) • The environment along the route affects my route choice	*Five point Likert scale (Strongly disagree to strongly agree)*
Travel route motivation	**Question 3 (Q3).** How important are the following when you choose your typical route in the SUMMER? Focus on when you WALK or CYCLE as part of your everyday travel (e.g. to go to work, school, shop but excluding leisure trips)? * Does this change over WINTER?	• The route is fast • The route is familiar to me • The route is pleasant (e.g. I like the views or sounds along the route) • The route goes through nature (e.g. trees and other vegetation, parks, along water) • The route is well-maintained (e.g. clean, good surface, well-lit) • The route is well-connected (the route infrastructure is continuous) • The route feels safe (e.g. from other traffic or people) • The route has variation and changing environment • The route is not crowded with people or traffic	*Five point Likert scale (Very unimportant to very important)*
Sensory aspects	**Question 4 (Q4).** How important are the following sensory aspects to your pleasant travel experience in the SUMMER? Focus on when you WALK or CYCLE as part of your everyday travel (e.g. to go to work, school, shop, but excluding leisure trips). * Does this change over WINTER?	• Aesthetics: The route is visually pleasant or appealing • Soundscape: The route is not noisy, so I can enjoy quietness or sounds of nature • Air quality: The air is clean along the route • Weather comfort: It feels comfortable in different weather conditions • Liveliness: The route is lively and/or has various services • Light: There is enough light along the route	*Five point Likert scale (Very unimportant to very important)*
Travel duration	**Question 5 (Q5).** In a typical day in SUMMER, what is the total duration (total minutes) of all your walking and/or cycling trips (e.g. to go to work, school, shop, but excluding leisure)? * Does this change over WINTER?	• 0 to 10 minutes • 10 to 30 minutes • 30 to 60 minutes • More than 60 minutes	*Select one option*
Longer travel duration	**Question 6 (Q6).** How much LONGER (in total minutes per day) would you be willing to walk and cycle for a greener route in SUMMER (e.g. more trees or other vegetation along the route) * Does this change over WINTER?	• I am not willing to walk or cycle longer even if the route is greener • 0 to 5 minutes • 5 to 10 minutes • 10-20 minutes • more than 20 minutes	*Select one option*

Section two opened by **Question 7 (Q7)** requesting respondents to identify their approximate home location (typical starting point for everyday active travel) by indicating their nearest street intersection. The home location data of Q7 is not provided in this dataset due to privacy reasons. Respondents were then asked in Q8 to map one or more places along their everyday cycling or walking routes that they perceived as pleasant or unpleasant. For each point marked, a pop-up box would invite respondents to allocate the point to given positive and negative exposure variables (refer Table 4) based on seasonal (summer/winter/both) and diurnal variation (daytime /after dark/both) [2]. The exposure options were matched with the sensory variables provided in Q4 (refer Table 3 previously). Additionally, in Q9 respondents could identify where to add street greenery along their everyday travel routes to improve their active travel experience. For each point marked, a pop-up box would ask respondents about what type of street greenery element they would recommend at the location. Each element was accompanied by an image to aid visualisation of the element by the respondent (refer Table 5). The street greenery elements were preselected based on reviewing urban planning documents and in consultation with urban and landscape planners at the City of Helsinki.

**Table 4:** Definition of pleasant and unpleasant exposure attributes in the PPGIS survey.

Question 8 (Q8) When you walk or cycle in your everyday travel (e.g. to go to work, school, shop), which places do you find PLEASANT or UNPLEASANT?** Please click and locate on the map one or more of the green and red buttons below to places that you find either PLEASANT or UNPLEASANT.	
Exposure attribute	Given definition
Pleasant	
*Aesthetics*	It is visually pleasant or appealing here
*Soundscape*	Here it is not noisy, so I can enjoy quietness or sounds of nature
*Air quality*	The air is clean here
*Weather comfort*	It feels comfortable here in different weather conditions
*Liveliness*	Here it is lively and/or there are various services
*Nature*	Here I am in or close to nature
Unpleasant	
*Unsafe*	I find it unsafe or scary here (e.g. because of traffic, people or animals)
*Noisy*	Here it is too noisy (e.g. from traffic, people or construction)
*No nature*	Here there is not enough natural elements (e.g. trees or other vegetation)
*Poor maintenance*	Here it is untidy or the walking or cycling infrastructure is absent or in poor condition
*Heavy traffic*	There is too much traffic here and it feels stressful
*Weather discomfort*	It feels uncomfortable here in different weather conditions

**Table 5:** Street greenery elements and accompanying images provided during the survey.

Question 9 (Q9) When you WALK or CYCLE in your everyday travel (e.g. to go to work, school, shop, but excluding leisure trips), are there any places you would like to add more street greenery? If not, skip this question.** Please click and locate on the map where and what type of green elements you would like to add.	
Street greenery element	Survey visual
Street tree	
Shrubs	
Vertical greenery	
Plant beds and parklets	
Lawns and meadows	
Seasonal flowers	

Finally, key socio-demographic background information was collected for each respondent as detailed in Table 1 previously. Most socio-demographic variables were consistent across the five study cities. The exceptions were mother tongue and average monthly income, which were customised to each city context by most common language spoken and income data derived from relevant national statistical databases, as described in [2].

Both survey datasets were cleaned and pre-processed to remove responses that fell outside of the study areas for each city, defined by level 1 classifications of Functional Urban Areas in European cities [10]. Data from surveys that were commenced and consent given, but closed by the respondent before the survey end, are included in the datasets.

## Limitations

There are limitations with the datasets as described in Korpilo et al. [2]. Pertaining specifically to the data shared in this article, it is important to note that survey questions focused on summer mobility may suffer from recall bias as the survey was distributed in the northern hemisphere winter. In addition, questions that focused on winter mobility received limited number of responses compared to summer (respondents were asked to answer the same question first for summer and then for winter). Moreover, a second survey round was required for Copenhagen in June (northern hemisphere summer), as described previously, leading to possible unbalanced effect of the recall bias in Copenhagen compared to other cities. However, the second sampling round occurred in Copenhagen only a few weeks after leaf-out of the trees, reducing the potential recall bias compared to the leafless sampling period in other cities. The data may also be subject to possible self-selection bias, with respondents more engaged in active travel on an everyday basis more likely to respond. The survey was distributed digitally, leading to possible exclusion by under-represented groups in digital and online spaces such as the elderly (indeed, there was slight underrepresentation of respondents aged 65+).

## Ethics Statement

The authors confirm that they abide by the ethical requirements for publication in Data in Brief and that the work presented here does not involve human subjects, animal experiments, or any data collected from social media platforms.

## CRediT Author Statement

**Rory Taylor:** data curation, project administration, visualisation, writing – original draft, writing – review and editing

**Silviya Korpilo:** methodology, investigation, data curation, writing – review and editing

**Elias Willberg:** methodology, investigation, data curation, writing – review and editing

**Kamyar Hasanzadeh:** writing – review and editing

**Robert Klein:** writing – review and editing

**Kerli Müürisepp:** writing – review and editing

**Anton Stahl Olafsson:** data curation, project administration, writing – review and editing

**Tuuli Toivonen:** conceptualisation, supervision, funding acquisition, writing – review and editing

## Data Availability

ZenodoGREENTRAVEL Public Participation GIS survey on active mobility in Europe (Original data)

## Appendix Supplementary materials

Image, application 1

Image, application 2

Image, application 3
