# Supplementary material for: Data from a public participation GIS survey on the everyday active travel experiences of residents from five European cities
Source: Data Brief. 2026 Jun 15;67:112981. doi: 10.1016/j.dib.2026.112981 (PMC13315897; doi:10.1016/j.dib.2026.112981)
Supplement: Supplementary file 3 [file mmc3.pdf]

GREENTRAVEL
A survey on your everyday active travel environment

Funded by the European Union

European Research Council
Established by the European Commission

HELSINGIN YLIOPISTO
HELSINGFORS UNIVERSITET
UNIVERSITY OF HELSINKI

Helsinki

ULPGC
Universidad de Las Palmas de Gran Canaria

Which city do you live in?

The GREENTRAVEL survey is conducted across five European cities. Before beginning the survey, please select the city in which you live.

Which city do you live in?

☐ Helsinki Metropolitan Area
☐ Greater London
☐ Munich
☐ Las Palmas
☐ Greater Copenhagen

Thank you for your interest in this survey!

The GREENTRAVEL survey asks what you perceive as important during your everyday active travel, how you experience greenery during that travel and how these may change in different seasons.

By *everyday active travel* we mean non-motorised forms of travel such as walking or cycling that you use on a regular daily basis to get to a destination (e.g. go to work, school, shop, but excluding trips for leisure). This could be used in combination with other forms of travel such as a personal vehicle or public transport. By answering this survey you can help us understand what contributes to a pleasant active travel environment and what is the role of greenery in it. This is important knowledge for improving travel routes and urban planning in general.

The survey is conducted by University of Helsinki, as part of the GREENTRAVEL Project (2023-2027) funded by the European Union (ERC, GREENTRAVEL, 101044906). Views and opinions expressed are however those of the authors only and do not necessarily reflect those of the European Union or the European Research Council Executive Agency. Neither the European Union nor the granting authority can be held responsible for them.

The survey comprises of three parts including three simple mapping tasks. **It should take approximately 15 minutes to complete**. We will collect, analyze, publish and store your data confidentially and anonymously in accordance with the General Data Protection Regulation of the European Union (see below for Data Protection Notice). ☎ If you have any questions regarding the survey, please email Dr. Silviya Korpilo at greentravel-project[at]helsinki.fi.

By continuing with this survey, you are agreeing that: (1) you understand the purpose of this research, (2) your participation is voluntary and (3) you are at least 18 years of age.

Do you agree to take part in this survey?

☐ I agree to take part in this survey managed by University of Helsinki.

GREENTRAVEL Data Protection Notice (English)

Understanding your daily travel

1. In the SUMMER, which travel modes do you use most in your everyday travel (e.g. to go to work, school, shop, but excluding trips for leisure)? Please rank up to three options according to time spent.

1

- ☐ Walking
- ☐ Cycling
- ☐ Electric bike
- ☐ Electric scooter
- ☐ Car
- ☐ Public transport

2

- ☐ Walking
- ☐ Cycling
- ☐ Electric bike
- ☐ Electric scooter
- ☐ Car
- ☐ Public transport

3

- ☐ Walking
- ☐ Cycling
- ☐ Electric bike
- ☐ Electric scooter
- ☐ Car
- ☐ Public transport

Does this change over WINTER?

2. Thinking about SUMMER, to what extent do you agree or disagree with the following statements about your everyday travel choices?

	Strongly disagree	Disagree	Neither agree/nor disagree	Agree	Strongly agree
The environment along my route affects whether I travel or not	◯	◯	◯	◯	◯
The environment along my route affects my transport mode choice (e.g. walk, cycle, car or public transport)	◯	◯	◯	◯	◯
The environment along the route	◯	◯	◯	◯	◯

affects my route choice					

Does this change over WINTER?

3. How important are the following when you choose your typical route in the SUMMER? Focus on when you WALK or CYCLE as part of your everyday travel (e.g. to go to work, school, shop but excluding leisure trips)?

	Very unimportant	Unimportant	Neither unimportant or important	Important	Very important
The route is fast	○	○	○	○	○
The route is familiar to me	○	○	○	○	○
The route is pleasant (e.g. I like the views or sounds along the route)	○	○	○	○	○
The route goes through nature (e.g. trees and other vegetation, parks, along water)	○	○	○	○	○
The route is well-maintained (e.g. clean, good surface, well-lit)	○	○	○	○	○
The route is well-connected (the route infrastructure is continuous)	○	○	○	○	○
The route feels safe (e.g. from other traffic or people)	○	○	○	○	○
The route has variation and changing environment	○	○	○	○	○
The route is not crowded with people or traffic	○	○	○	○	○

Does this change over WINTER?

Your daily travel environment

4. How important are the following sensory aspects to your pleasant travel experience in the SUMMER? Focus on when you WALK or CYCLE as part of your everyday travel (e.g. to go to work, school, shop, but excluding leisure trips).

	Very unimportant	Unimportant	Neither unimportant or important	Important	Very important
AESTHETICS: the route is visually pleasant or appealing	○	○	○	○	○
SOUNDSCAPE: the route is not noisy, so I can enjoy quietness or sounds of nature	○	○	○	○	○
AIR QUALITY: the air is clean along the route	○	○	○	○	○
WEATHER COMFORT: it feels comfortable in different weather conditions	○	○	○	○	○
LIVELINESS: the route is lively and/or has various services	○	○	○	○	○
LIGHT: there is enough light along the route	○	○	○	○	○

Does this change over WINTER?

5. In a typical day in SUMMER, what is the total duration (total minutes) of all your walking and/or cycling trips (e.g. to go to work, school, shop, but excluding leisure)?

- ☐ 0 to 10 minutes
- ☐ 10 to 30 minutes
- ☐ 30 to 60 minutes
- ☐ More than 60 minutes

Does this change over WINTER?

6. How much LONGER (in total minutes per day) would you be willing to walk and cycle for a greener route in SUMMER (e.g. more trees or other vegetation along the route)?

- ☐ I am not willing to walk or cycle longer even if the route is greener
- ☐ 0 to 5 minutes
- ☐ 5 to 10 minutes
- ☐ 10-20 minutes
- ☐ more than 20 minutes

Does this change over WINTER?

Your neighbourhood

Here we ask about your approximate home location. This is to help us understand the general location in which you start your typical travel routes. We will analyse home data for all respondents collectively, so no individual home locations will be examined separately. You don't need to locate the exact location of your home. Instead please use the blue button below to mark a street intersection closest to you home.

You can zoom in and out of the map as needed. You can also change the background to a topographic map by clicking on the blue figure in the top right corner. *TIP! You can remove any marker you've placed by clicking on it and selecting 'Delete'*.

Mapping places along your daily active travel routes

8. When you walk or cycle in your everyday travel (e.g. to go to work, school, shop), which places do you find PLEASANT or UNPLEASANT? Please click and locate on the map one or more of the green and red buttons below to places that you find either PLEASANT or UNPLEASANT.

9. When you WALK or CYCLE in your everyday travel (e.g. to go to work, school, shop, but excluding leisure trips), are there any places you would like to add more street greenery? If not, skip this question. Please click and locate on the map where and what type of green elements you would like to add.

Your background

10. On a scale of 1 to 10, how busy do you consider your everyday life to be at the moment?

Enter a value between 1 (Not at all busy) and 10 (Extremely busy)

11. To which gender do you most identify with?

☐ Female
☐ Male
☐ Other
☐ Prefer not to say

12. To which age group do you belong?

☐ 18 – 24
☐ 25 – 34
☐ 35 – 44
☐ 45 – 54
☐ 55 – 64
☐ 65+

13. What is your mother tongue? You can choose multiple options.

☐ Finnish
☐ Swedish
☐ English
☐ Russian
☐ Estonian
☐ Somali
☐ Arabic
☐ Mandarin
☐ Other

Other (please specify)

14. How many persons live in your household (including yourself)?

Value cannot be below 1

15. How many of them are under 18? (number of children)

Value cannot be below 0

16. What is your current employment status?

☐ In full-time paid work (employee, self-employed, working for your family business)

☐ In part-time paid work
☐ Unemployed
☐ Student
☐ In community or military service
☐ Stay at home, looking after children or other persons
☐ Retired
☐ Permanently sick or disabled

Other (please specify)

17. What is the highest level of formal education you have completed? Please tick one most appropriate response

☐ No formal schooling completed
☐ Upper secondary education
☐ Trade/Technical/vocational training
☐ Bachelor’s degree
☐ Master’s degree
☐ Doctoral degree

18. What is your average monthly net income (after taxes)? Please mark your estimation if you're unsure of the exact amount.

☐ Less than 1,000 euros
☐ 1,000 to 2,000 euros
☐ 2,001 to 3,000 euros
☐ 3,001 to 4,000 euros
☐ More than 4,000 euros

You've reached the end of the survey! Are you happy with all your answers?

☐ Yes, I am.

Thank you for taking part in this survey!

Would you like a copy of the survey results? If yes, please email the GREENTRAVEL team at greentravel-project[at]helsinki.fi.

Would you like to provide any feedback on this survey?
